# Outer Membrane Vesicle Production by *Helicobacter pylori* Represents an Approach for the Delivery of Virulence Factors CagA, VacA and UreA into Human Gastric Adenocarcinoma (AGS) Cells

**DOI:** 10.3390/ijms22083942

**Published:** 2021-04-11

**Authors:** Yongyu Chew, Hsin-Yu Chung, Po-Yi Lin, Deng-Chyang Wu, Shau-Ku Huang, Mou-Chieh Kao

**Affiliations:** 1 Institute of Molecular Medicine, National Tsing Hua University, Hsinchu 30013, Taiwan; jynn1992@gapp.nthu.edu.tw (Y.C.); angelfriends1115@gmail.com (H.-Y.C.); likecomputerqoo@hotmail.com (P.-Y.L.); 2 Division of Gastroenterology, Department of Internal Medicine, Kaoshiung Medical University Hospital, Kaoshiung Medical University, Kaoshiung 80756, Taiwan; dechwu@yahoo.com; 3 National Institute of Environmental Health Sciences, National Health Research Institutes, Zhunan 35053, Taiwan; skhuang@nhri.org.tw; 4 Department of Life Science, College of Life Science, National Tsing Hua University, Hsinchu 30013, Taiwan

**Keywords:** *Helicobacter pylori*, outer membrane vesicles, CagA, VacA, UreA, gastrointestinal disorders

## Abstract

*Helicobacter pylori* infection is the etiology of several gastric-related diseases including gastric cancer. Cytotoxin associated gene A (CagA), vacuolating cytotoxin A (VacA) and α-subunit of urease (UreA) are three major virulence factors of *H. pylori*, and each of them has a distinct entry pathway and pathogenic mechanism during bacterial infection. *H. pylori* can shed outer membrane vesicles (OMVs). Therefore, it would be interesting to explore the production kinetics of *H. pylori* OMVs and its connection with the entry of key virulence factors into host cells. Here, we isolated OMVs from *H. pylori* 26,695 strain and characterized their properties and interaction kinetics with human gastric adenocarcinoma (AGS) cells. We found that the generation of OMVs and the presence of CagA, VacA and UreA in OMVs were a lasting event throughout different phases of bacterial growth. *H. pylori* OMVs entered AGS cells mainly through macropinocytosis/phagocytosis. Furthermore, CagA, VacA and UreA could enter AGS cells via OMVs and the treatment with *H. pylori* OMVs would cause cell death. Comparison of *H. pylori* 26,695 and clinical strains suggested that the production and characteristics of OMVs are not only limited to laboratory strains commonly in use, but a general phenomenon to most *H. pylori* strains.

## 1. Introduction

*Helicobacter pylori* is a flagellate spiral-shaped Gram-negative microaerophilic bacterium which is associated with several upper gastrointestinal disorders, such as gastritis, peptic ulcer disease, gastric mucosa-associated lymphoid tissue (MALT) lymphoma, and even gastric cancer [1]. During infection, *H. pylori* releases several virulence factors such as cytotoxin-associated gene A (CagA), vacuolating cytotoxin (VacA) and urease, whose corresponding genes are frequently found in strains with enhanced pathogenicity. CagA is the best characterized virulence factor in *H. pylori*. It is an approximately 140-kDa immune-dominant protein encoded by the *cagA* gene localized at the cag pathogenicity island (PAI) that encodes most of the components of the Type IV secretion system (T4SS) [2]. When *H. pylori* attaches to the epithelial cells of host gastric tissues, CagA is rapidly injected into the cells through T4SS and phosphorylated by host c-Src family kinases at the tyrosine residue of its multiple Glu-Pro-Ile-Tyr-Ala (EPIYA) sites [3,4,5,6]. The phosphorylated CagA binds an eukaryotic phosphatase SHP-2 and induces the cytoskeleton remodeling, which causes the elongation of gastric epithelial cells, known as the hummingbird phenotype [6]. Since CagA activates several signaling cascades associated with carcinogenesis, it has been termed a “bacterial oncoprotein” [7]. VacA is another key virulence factor and present essentially in all *H. pylori* strains. However, it is not always active as an in vitro vacuolating toxin because of the existence of sequence variation [8]. VacA is first synthesized as a 140-kDa precursor and then secreted as an 88-kDa protein. Secreted VacA can form an oligomeric structure which binds to the host cell surface and then enters host cells by lipid raft-dependent, clathrin-independent endocytosis [9,10]. After internalization, VacA forms an anion selective channel on the late endosomes and enhances vacuolar-type H^+^-ATPase (V-ATPase) proton pump activity, causing acidification of endosomes. Osmotically weak bases such as ammonia and water will diffuse into the late endosomes, resulting in the swelling of endosomes [11,12]. Research has shown that VacA located at the endosomal membrane can be transferred to mitochondria by an endosome–mitochondrion juxtaposition in a BAX/BAK-dependent manner [13]. Insertion of VacA to mitochondria might lead to hyperpolarization, resulting in the inhibition of mitochondrial functions and the loss of mitochondrial membrane potential [14]. These effects will cause the release of cytochrome *c* from mitochondria and subsequent activation of downstream caspase activities that finally lead to apoptosis [15]. During *H. pylori* infection, the bacterium can adapt to the acidic gastric environment with the help of urease enzyme [16]. The urease enzyme is made up by a 27-kDa α-subunit (UreA) and a 62-kDa β-subunit (UreB), and can hydrolyze urea to ammonia and bicarbonate [17]. Ammonia produced by urease can neutralize the acidic environment and also cause damage to the epithelial cells, such as swelling of acidic intracellular compartments, alterations of vesicular membrane transport, repression of protein synthesis and ATP production, and even cell-cycle arrest [16]. The production of bicarbonate was found to suppress the bactericidal effect of nitric oxide metabolite, known as peroxynitrite, suggesting that the urease enzyme not only promotes the survival of bacteria but also has a protective effect on *H. pylori* [18].

Gram-negative bacteria are able to shed vesicles from their outer membrane surface [19,20,21,22,23,24,25,26]. These 20–300-nm vesicles produced are surrounded by an outer membrane layer, containing lipopolysaccharide (LPS) and also outer membrane proteins (OMPs), and some vesicles might also contain the periplasmic and cytoplasmic proteins from the bacteria [27]. Outer membrane vesicles (OMVs) are thought to play roles in promoting bacterial survival, bacterial communication, delivering toxins and virulence factors, modulating host immune response and transferring genetic materials [28,29]. Therefore, the OMVs of disease-associated bacteria may have important implications on the pathogenesis of bacterial infection. Numerous studies targeting OMVs focus on the delivery of toxins and interactions with host cells. Some examples of toxin delivery through OMVs are enterotoxigenic *Escherichia coli* (ETEC) toxins such as heat-labile toxin (LT) [19], cytotoxic necrotizing factor 1 toxin (CNF1) [20], α-haemolysin (HlyA) [21], Shiga toxin 2a (Stx2a), cytolethal distending toxin V (CdtV) and flagellin [22], *Campylobacter jejuni* cytolethal distending toxin (CDT) [23], *Salmonella typhi* ClyA cytotoxin [24], *Neisseria gonorrhoeae PorB protein* [25] and *Fusobacterium nucleatum FadA* [26]. *H. pylori* OMVs are proven to contain adhesins [30,31,32], LPS and the major virulence factors CagA [30,31,33], VacA [30,31,32,33,34], and UreA [30,31,32]. Therefore, they might represent another approach to deliver these toxins and virulence factors to the gastric epithelium. They also potentially play a role in the pathogenesis of *H. pylori* infection, such as triggering the production of cytokines and inducing apoptosis in gastric epithelial cells [35,36]. Gastric epithelial cells can take up *H. pylori* OMVs by endocytosis, which can be mediated by either clathrin-dependent or clathrin-independent pathways and is enhanced by VacA protein [37,38].

In this study, we tried to characterize the biochemical and biophysical properties of OMVs and reveal the potential role of OMVs released from *H. pylori* and their involvement in *H. pylori* infection. We first isolated OMVs from *H. pylori* 26,695 strain at different time points of bacterial growth and confirmed that the generation of OMVs and the presence of several virulence factors in OMVs were a lasting event throughout different phases of *H. pylori* growth. CagA was found to be weakly associated with OMVs, but VacA had a strong association with OMVs, or was even inserted in the membrane or located in the lumen of OMVs. We also revealed that *H. pylori* OMVs entered human gastric adenocarcinoma (AGS) cells mainly through macropinocytosis/phagocytosis. The virulence factors CagA, VacA and UreA can be delivered into AGS cells via OMVs and the treatment with *H. pylori* OMVs could cause a decrease in cell viability. Comparison of *H. pylori* 26,695 and clinical strains suggested that the production and characteristics of OMVs were not only limited to laboratory strains commonly in use, but a general phenomenon to most *H. pylori* strains.

## 2. Results

### 2.1. Identification and Characterization of OMVs Isolated from H. pylori 26,695 Strain

**Figure 1:**
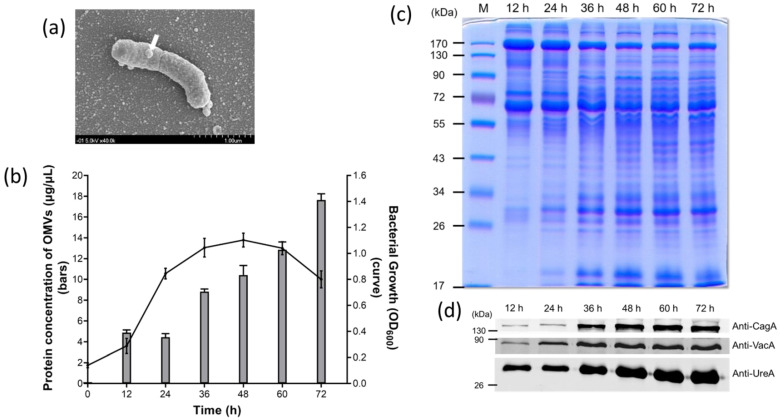
Identification and characterization of outer membrane vesicles (OMVs) isolated from *H. pylori* 26,695 strain. (**a**) The scanning election micrograph of *H. pylori* 26,695 strain taken with 40,000 × magnification. The white arrow indicates that an OMV is being produced from the surface of a bacterium. (**b**) The growth curve of *H. pylori* 26,695 strain and the corresponding protein concentration of OMVs isolated during the course of bacterial growth. Data are shown as the mean ± SD (*n* = 3). (**c**) Protein profiles of OMVs isolated from *H. pylori* 26,695 strain at different time points of culture. M: prestained protein markers. (**d**) The presence of key virulence factors in OMVs isolated from *H. pylori* 26,695 strain at different time points.

Under a scanning electron microscopy (SEM), OMVs were observed to originate from the cell surface envelope of *H. pylori*, and appeared as spheroid particles with a size of approximately 10 to 300 nm in diameter (Figure 1a). To investigate the yield of OMVs during different phases of *H. pylori* growth, bacterial culture was first monitored by measuring the optical density at OD_600_ at different time points, and the protein level of OMVs collected at the corresponding time point was determined (Figure 1b). *H. pylori* 26,695 strain showed a typical pattern of bacterial growth curve with the lag phase between 0 and 12 h, the log phase between 12 and 36 h, the stationary phase between 36 and 60 h, and entered the death phase after 60 h of growth. Interestingly, the production of OMVs isolated from *H. pylori* 26,695 strain significantly increased during the stationary phase of growth and continued to accumulate even at the early death phase.

Comparison of the protein profiles of OMVs isolated from different time points revealed that the presence and the amount of certain proteins within OMVs were different at different stages of *H. pylori* growth (Figure 1c). In the early stage of bacterial growth, OMVs contained a smaller amount of low molecular weight proteins. The low molecular weight proteins were increased considerably after 36 h of growth and maintained a plateau thereafter. Interestingly, the three major virulence proteins tested, including CagA, VacA and UreA, were observed in OMVs prepared from the culture supernatant of all growth phases but most significant after 36 h of growth (Figure 1d). These results indicated that the protein compositions of OMVs, especially the three virulence factors tested, did not have a dramatic change after the late log phase of *H. pylori* growth. By considering the result of OMV production together, we selected 60 h of bacterial growth as the time point for isolation of OMVs. At this time point, bacteria were viable, contained a lower level of lysed cell debris and produced sufficient quantities of vesicles for later studies. The size distribution of OMVs isolated at 60 h of *H. pylori* growth was analyzed using dynamic light scattering. The OMVs isolated from *H. pylori* 26,695 strain ranged from 50 to 400 nm in diameter, with an average diameter of 120.20 ± 39.06 nm (Table 1).

### 2.2. The Association of Key Virulence Factors with OMVs Isolated from H. pylori 26,695 Strain

**Figure 2:**
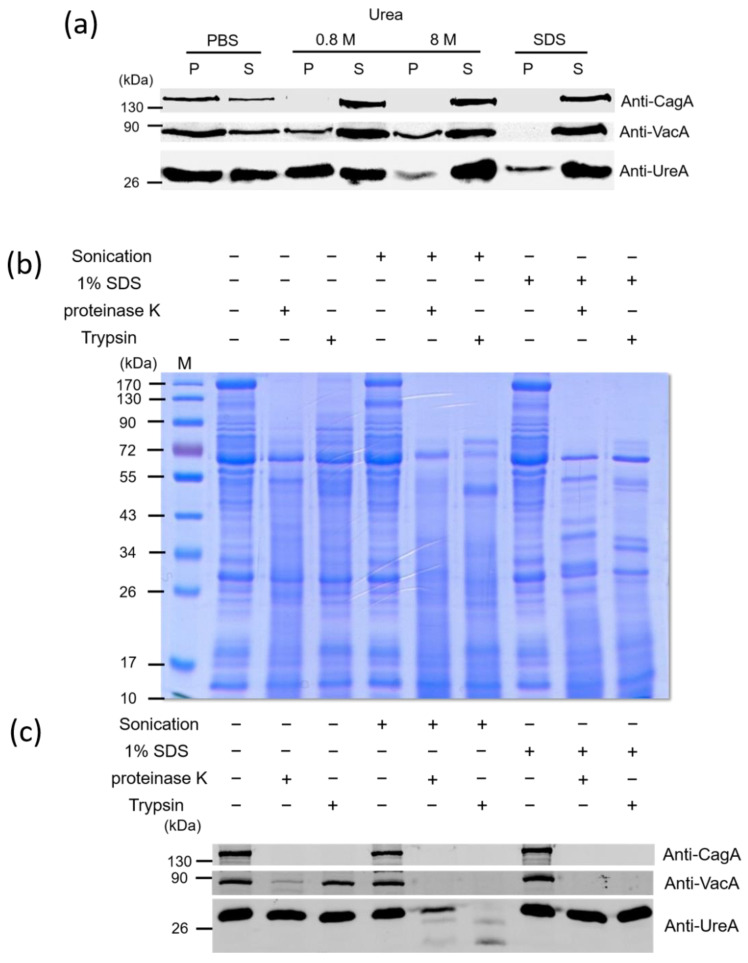
The association of key virulence factors with OMVs isolated from *H. pylori* 26,695 strain. OMVs isolated from *H. pylori* 26,695 strain were treated with (**a**) PBS, 0.8 M, 8 M urea or 1% SDS, (**b**,**c**) sonication or 1% SDS, followed by proteinase K or trypsin treatment. The obtained protein samples were then analyzed by 10% SDS-PAGE, followed with Commasie blue staining (**b**) or immunoblotting detection (**a**,**c**) with anti-CagA, anti-VacA and anti-UreA antibodies. M: prestained protein markers.

We next tried to investigate the association and location of the virulence factors in OMVs. OMVs isolated from *H. pylori* 26,695 strain were treated with phosphate buffer solution (PBS), different concentrations of urea or SDS, followed by centrifugation. As shown in Figure 2a, after 0.8 M urea treatment, CagA was all present in the supernatants. In contrast, some of VacA was still present in the pellets even treated with 8 M urea. However, this toxin was completely removed from OMVs after 1% SDS treatment. Interestingly, all the above treatments could not completely remove UreA from OMVs. To further explore the association between major virulence factors and OMVs, OMVs were also treated with proteinase K or trypsin in the absence or presence of 1% SDS or after sonication (Figure 2b,c). Sonication and the addition of SDS both disrupted the integrity of OMVs but with different degrees, allowing access of the protease to the interior contents of the vesicles. Incubation of purified OMVs with proteinase K or trypsin alone all resulted in some loss of a great number of presumably surface-exposed proteins, but the overall protein profiles of OMVs treated with these two proteases were similar but not identical (Figure 2b). In addition, incubation with either one of the proteases after sonication or in the presence of SDS resulted in a greater loss of protein bands, suggesting that the remaining proteins are resistant to protease digestion under current experimental conditions. The result from immunoblotting analysis also indicated that VacA and UreA were partly protected from proteinase K or trypsin digestion when vesicles were incubated with either of these two proteases alone (Figure 2c). However, CagA was protected weakly with these treatments. In contrast, CagA and VacA were completely degraded by proteinase K or trypsin treatment in the presence of SDS or when previously treated with sonication. The above results suggested that CagA protein is loosely associated with the surface of OMVs isolated from *H. pylori* 26,695 strain, and VacA could be tightly associated with the surface of OMVs, inserted in the membrane of OMVs or located in the lumen of OMVs. In addition, since UreA was still detectable after 1% SDS treatment or sonication followed by protease digestion treatment, the exact association of UreA with OMVs isolated from *H. pylori* 26,695 strain is not conclusive and deserved further investigation.

### 2.3. OMVs Isolated from H. pylori 26,695 Strain Were Associated with and Internalized into AGS Cells

**Figure 3:**
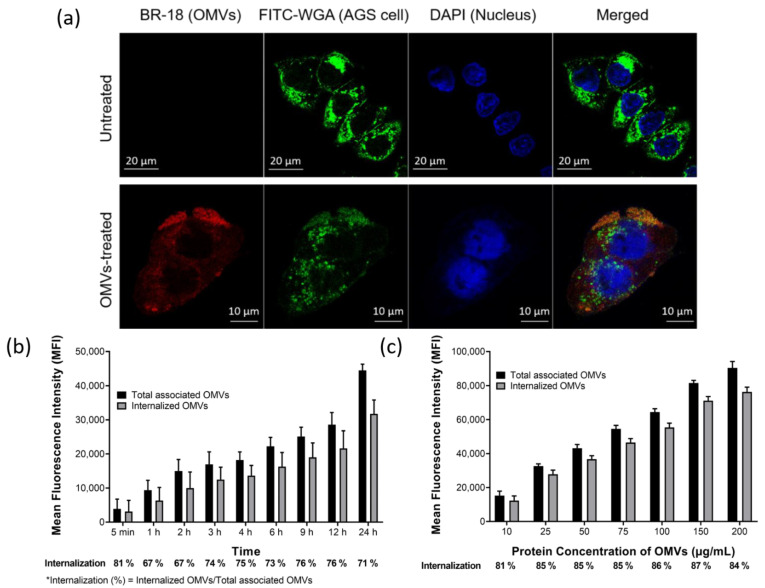
OMVs isolated from *H. pylori* 26,695 strain were associated and internalized into human gastric adenocarcinoma (AGS) cells. (**a**) The association and internalization of OMVs with AGS cells after 1 h of co-incubation were analyzed using LSM510 confocal microscopy. Green: AGS cells labeled with fluorescein isothiocyanate (FITC)-wheat germ agglutinin (WGA); red: BR-18-labeled OMVs isolated from *H. pylori* 26,695 strain; blue: 4′,6-Diamidino-2-Phenylindole (DAPI) stained nuclei. The (**b**) time- and (**c**) dose-dependent association and internalization of BR-18-labeled OMVs isolated from *H. pylori* 26,695 strain with AGS cells. The addition of trypan blue (final concentration of 0.025%) was used to distinguish the internalized OMVs from the cell-bound but non-internalized OMVs. Data are shown as the mean ± SD (*n* = 3).

To explore if OMVs could be associated with and then enter AGS cells, the confocal microscopy approach was used in this study. AGS cells were pre-incubated with fluorescein isothiocyanate (FITC)-wheat germ agglutinin (WGA) which was utilized for fluorescent detection of eukaryotic cells. The FITC-WGA labeled AGS cells were then treated with or without BR-18-labeled OMVs for 1 h. The resulting images showed that most of BR-18-labeled OMVs were found to be located around or within AGS cells, while no OMV signal was observed in untreated cells (Figure 3a). To further investigate the adherence and internalization kinetics of OMVs to epithelial cells, AGS cells were first incubated with BR-18-labeled OMVs and trypan blue was then used to distinguish the internalized OMVs from the cell-bound but non-internalized OMVs since the extracellular fluorescence from the extracellular BR-18-labeled OMVs would be quenched by trypan blue treatment. The result showed that BR-18-labeled OMVs were adhered to AGS cells within 5 min of incubation, and the OMV–cell association steadily increased up to 24 h (Figure 3b). Similarly, the amount of internalized OMVs, which was represented by the fluorescent signal not quenched by the addition of trypan blue, was also increased with the time of AGS–OMV co-incubation. In addition, except at the first 5 min, the proportions of internalized OMVs to total associated OMVs were also steadily increased with time and then maintained around 71–76% in cells for 24 h of the experimental period. Furthermore, AGS cells were also incubated with increasing concentrations of BR-18-labeled OMVs to evaluate the dose effect on the association and internalization of OMVs (Figure 3c). The OMV–AGS cell association increased in a dose-dependent manner after co-incubation for 24 h. The proportions of internalized OMVs to total associated OMVs were all around 80% for different concentrations of OMVs used. These results suggested that the internalization of OMVs into AGS cells is a time-dependent and dose-independent process. These results demonstrated that OMVs isolated from *H. pylori* 26,695 strain could adhere to and be internalized by AGS cells.

### 2.4. OMVs Isolated from H. pylori 26,695 Strain Entered AGS Cells Mainly by Macropinocytosis/Phagocytosis

**Figure 4:**
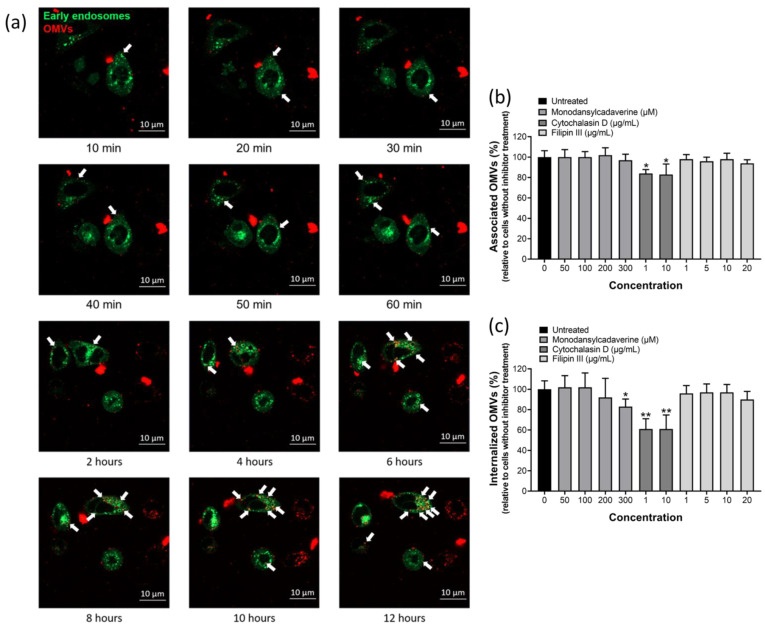
OMVs isolated from *H. pylori* 26,695 strain entered AGS cells mainly by macropinocytosis/phagocytosis. (**a**) The association of OMVs with early endosomes after co-incubation of AGS cells with 1,1′-diocatadecyl-3,3,3′,3′-tetramethylindocar-bocyanine perchlorate (DiI)-labeled OMVs for up to 12 h weas analyzed using LSM800 confocal microscopy. Green: early endosomes-GFP; red: DiI-labeled OMVs isolated from *H. pylori* 26,695 strain. White arrows indicate OMVs that were in close contact with early endosomes. The effect of endocytic inhibitors on the (**b**) association and (**c**) internalization of BR-18-labeled OMVs isolated from *H. pylori* 26,695 strain with AGS cells. Data are shown as the mean ± SD (*n* = 3, * *p* < 0.05; ** *p* < 0.01).

We next tried to explore the mechanism whereby OMVs deliver bacterial proteins into host cells. Several recent studies on other bacteria showed that proteins or toxins carried by OMVs could be delivered to the cytoplasm of host cells via fusion of the vesicles with the plasma membrane or through endocytosis [38,39,40]. OMVs were first labeled with 1,1′-diocatadecyl-3,3,3′,3′-tetramethylindocar-bocyanine perchlorate (DiI) and then added to AGS cells that were pre-labeled with early endosome-GFP (Figure 4a). DiI-labeled OMVs were found in AGS cells and were in close contact with early endosomes within 10 min of OMVs co-incubation and the amount of OMVs associated with early endosomes increased with time, suggesting that OMVs enter the cells by endocytosis. This phenomenon remained observable for at least 12 h of co-incubation. To investigate which endocytic pathways is involved in the adherence and internalization of OMVs isolated from *H. pylori* 26,695 strain, three inhibitors targeting different endocytotic pathways were used in this study. Monodansylcadaverine (MDC), Cytochalasin D and Filipin III were used to inhibit clathrin-mediated endocytosis, micropinocytosis/phagocytosis and lipid rafts/caveolae-mediated endocytosis, respectively (Figure 4b,c). Pretreatment of AGS cells with MDC or Filipin III did not affect the association of OMVs with AGS cells, but pretreatment with cytochalasin D caused a slight decrease in OMV–AGS cell association (Figure 4b). When trypan blue was added to detect the quantity of internalized OMVs, the result indicated that only AGS cells pretreated with cytochalasin D or a higher dosage of MDC (300 μM) significantly inhibited the internalization of OMVs, but the internalization level of BR-18-labeled OMVs in the present of Filipin III or low dosage of MDC had no significant difference (Figure 4c). The above findings suggested that macropinocytosis/phagocytosis is the main mechanism involved in the internalization of OMVs isolated from *H. pylori* 26,695 strain into AGS cells.

### 2.5. H. pylori OMVs Caused Cell Death after Co-Incubation with Gastric AGS Cells

**Figure 5:**
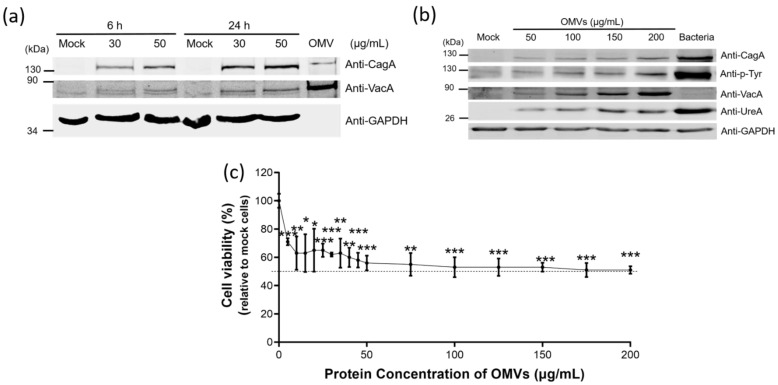
*H. pylori* OMVs caused cell death after co-incubation with gastric AGS cells. (**a**,**b**) OMVs-associated CagA, VacA and UreA were accumulated in the whole cell extracts of AGS cells after the co-incubation study. (**a**) Whole cell lysates from AGS cells co-incubated with OMVs isolated from *H. pylori* 26,695 strain for 6 h or 24 h. Mock, a control group without OMVs co-incubation. (**b**) Whole cell lysates from AGS cells treated with different concentrations of OMVs isolated from *H. pylori* 26,695 strain for 24 h or with bacteria of *H. pylori* 26,695 strain (multiplicity of infection (MOI) = 100) for 6 h. (**c**) The dose-dependent effect of OMVs isolated from *H. pylori* 26,695 strain on cell viability. Data are shown as the mean ± SD (*n* = 3, * *p* < 0.05; ** *p* < 0.01; *** *p* < 0.001).

Since we have demonstrated that *H. pylori* OMVs could carry three key virulence factors CagA, VacA and UreA, we next tried to investigate whether these three virulence factors could enter the host cells though the association with OMVs. Different concentrations of OMVs isolated from *H. pylori* 26,695 strain were co-incubated with AGS cells for 6 or 24 h. After thorough washing, the entry of CagA, VacA and UreA was evaluated on the whole cell lysates by immunoblotting with anti-CagA, anti-VacA and anti-UreA antibodies (Figure 5a,b). Both OMV-associated CagA and VacA could enter AGS cells in a dose- and time-dependent manner, revealing that these two virulence factors associated with OMVs could enter AGS cells successfully without the existence of bacterial *H. pylori*. Similar to *H. pylori* infection, the OMV-associated CagA was phophorylated after it entered AGS cells (Figure 5b), suggesting that CagA might be located at the surface of OMVs or might be released into the cytoplasm following its entry into AGS cells through OMVs. Interestingly, although CagA and VacA were both present in the bacteria and OMVs, their amount in infected cells was different. The amount of CagA in AGS cells was much less in OMVs treated cells compared to bacterial infection but VacA was much less in cells with bacterial infection compared to OMVs treatment. As for UreA, this urease subunit could enter AGS cells through OMVs in a dose-dependent manner (Figure 5b). Thus, the formation of OMVs represents as another approach for the entry of CagA, VacA and UreA into host cells during *H. pylori* infection, in addition to their original, well-recognized pathways for delivery.

We further analyzed the effect of OMVs treatment on the viability of AGS cells incubated with different concentrations of OMVs isolated from *H. pylori* 26,695 strain (Figure 5c). The cell viability was decreased along with the increasing concentration of OMVs. The median lethal dose (LD_50_) was around 100 μg/mL of OMVs co-incubation. Cell viability also reached a plateau after treatment with 100 μg/mL of OMVs. Co-incubation of AGS cells with a higher concentration of OMVs did not cause a further decrease in cell viability.

### 2.6. H. pylori Clinical Strains Produced OMVs with Characteristics Similar but Not Identical to Those of the 26,695 Strain

**Figure 6:**
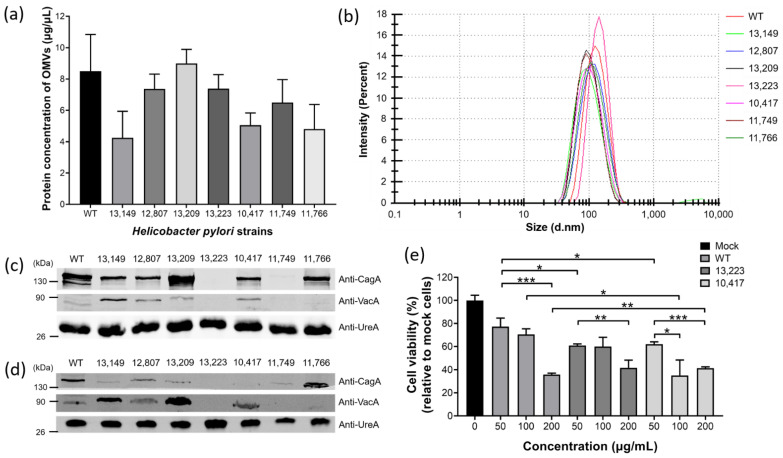
*H. pylori* clinical strains produced OMVs with characteristics similar but not identical to those of *H. pylori* 26,695 strain. (**a**) The production level of OMVs isolated from *H. pylori* 26,695 and various clinical strains. Data are shown as the mean ± SD (*n* = 3). (**b**) Particle size analysis of OMVs isolated from *H. pylori* 26,695 and various clinical strains. The presence of key virulence factors CagA, VacA and UreA in (**c**) whole bacterial lysates and (**d**) OMVs isolated from *H. pylori* 26,695 and various clinical strains. (**e**) The effect of OMVs isolated from *H. pylori* 26,695 and various clinical strains on the viability of AGS cells. Data are shown as the mean ± SD (*n* = 3, * *p* < 0.05; ** *p* < 0.01; *** *p* < 0.001).

To investigate if OMVs are also produced in clinical strains and possess similar characteristics to those of the laboratory strains, *H. pylori* 26,695 and various clinical strains isolated from patients with different gastrointestinal disorders, such as gastritis (13,149 strain), gastric ulcer (12,807 strain), duodenal ulcer (13,209 strain), gastric mucosa-associated lymphoid tissue (MALT) lymphoma (13,223 strain), and gastric adenocarcinoma grade I A (10,417 strain), III B (11,749 strain), and I B (11,766 strain) were harvested after 60 h of bacterial growth for isolation of OMVs. OMVs were successfully isolated from all tested strains. Although the protein concentration of OMVs isolated from an equal number of bacteria varied, there were no significant differences among all strains tested (Figure 6a). The sizes of OMVs isolated from all tested *H. pylori* strains ranged from 30 nm to 400 nm (Figure 6b), with an average diameter of 89 nm to 127 nm (Table 1).

Protein electrophoresis and immunoblotting analysis were also performed to analyze the protein contents, mainly key virulence factors CagA, VacA and UreA in bacteria and OMVs of *H. pylori* 26,695 and various clinical strains (Figure 6c,d). The results showed that different amounts of CagA and VacA were present in the bacteria and OMVs of most *H. pylori* strains tested, while UreA was present in all *H. pylori* strains in a similar amount. Interestingly, we observed that *H. pylori* clinical strains isolated from patients with much more severe diseases such as MALT lymphoma (13,223 strain) and gastric adenocarcinoma grade III B (11,749 strain) did not contain (or produced much less) the virulence factors CagA and VacA. To explore whether the presence of these virulence factors would affect the pathogenicity of the OMVs, AGS cells were incubated with different concentrations of OMVs isolated from *H. pylori* 26,695 strain, 13,223 strain (MALT lymphoma) and 10,417 strain (gastric adenocarcinoma grade I A), and the cell viability was analyzed (Figure 6e). The result showed that all tested strains caused cell death in a dose-dependent manner. Comparing the result of AGS cells co-incubated with 50 μg/mL of OMVs, OMVs derived from both 13,223 strain and 10,417 strain of clinical origins caused a significantly higher ratio of cell death than that from 26,695 strain. The above findings suggested that in addition to the virulence factors CagA and VacA, there might be other factors that affect the pathogenicity of *H. pylori* OMVs. Taken together, these results showed that *H. pylori* from different sources can produce OMVs, and the statuses of presence of virulence factors CagA, VacA and UreA in OMVs mostly correspond to those in bacteria. Therefore, the production of OMVs and their characteristics are not restricted to the commonly used *H. pylori* laboratory strain but also applicable to clinically isolated strains.

## 3. Discussion

*H. pylori* is a unique bacterium because it can successfully survive in an extremely acidic environment and coexist with humans to establish a dynamic balance within the stomach of humans for many years. Many reports have suggested that a complex interplay between *H. pylori* and host factors has evolved many bacterial factors, and some of them, including a number of adherence factors and virulence factors involved in the signaling pathways of host cells, have been identified by the scientific efforts made during the past several decades. However, the exact pathophysiological mechanisms of many gastric diseases are still not fully understood. Recent progress in this area has revealed that OMVs, which are constantly released from the surface of Gram-negative bacteria, can provide an additional mechanism for the pathogenicity of bacterial infection as they have the potential to cross the gastric epithelial barrier and may mediate delivery of virulence factors to host immune cells [41,42].

In this study, we have characterized OMVs isolated from *H. pylori* 26,695 strain and our findings are supported by several proteomic reports that CagA, VacA and UreA are indeed components of *H. pylori* OMVs [30,31,32]. According to one report, the OMV-associated VacA accounts only 25% of total VacA in the supernatant, while the remaining 75% is represented by free-soluble form of VacA [43]. It is still unknown if these two different forms of VacA possess distinct biological activities once having gained entry into host cells. Since CagA is also found to be associated with OMVs isolated from *H. pylori* 26,695 strain, OMVs might represent an alternative approach for *H. pylori* to transport CagA directly into host cells without the need for the assembly of a functional type IV secretion system. In addition, UreA, a subunit of the urease enzyme, is identified in *H. pylori* OMVs as well. Although the appearance of UreB, which can cooperate with UreA to form a functional urease hexamer, is not tested in our current study, it is highly possible this key urease subunit is also present in OMVs isolated from *H. pylori* 26,695 strain, just like other reported *H. pylori* strains (Culture Collection, University of Göteborg (CCUG) 17,875, J99 and National Collection of Type Cultures (NCTC)11,637) [30,31]. If these two subunits of urease can form a functional complex in OMVs, they may be capable of increasing pH around the parent bacteria to assist *H. pylori* colonization.

In our present study we could clearly detect CagA, VacA and UreA in OMVs of *H. pylori* 26,695 strain. However, OMV-associated CagA was not detected from the shedding cag-PAI^+^ 60,190 strain and scarcely detected in NCTC 11,637 strain [31,44], while VacA was barely detected in OMVs of *H. pylori* NCTC 11,637 strain. Similarly, only a very low level of urease activity was detected in the OMVs isolated from *H. pylori* strains 84–183, Tx-30a and 60,290 [44]. These differences strongly suggest that the presence and the amount of virulence factors associated with OMVs depend greatly on *H. pylori* strains.

To study the association of CagA, VacA and UreA with OMVs, we conducted two-well adopted dissociation and proteinase digestion assays to differentiate the loose-associated from the tight-associated virulence factors on OMVs. Based on the results, we conclude that CagA protein is located only on the surface of the OMVs isolated from *H. pylori* 26,695 strain, which agrees with the suggestion made by one previous report [30]. On the other hand, VacA could be tightly associated on the surface of OMVs, inserted in the membrane of OMVs or even located in the lumen of OMVs, which is also consistent with the TEM analysis from previous findings [45]. Interestingly, after treating *H. pylori* OMVs with a buffer containing 1% SDS or sonication, a portion of the UreA was still resistant to proteinase K digestion, indicating that UreA is tightly associated with OMVs or may exist as an insoluble structure under the above treatment conditions. Further experiments are required to unequivocally identify the association and location of the UreA subunit in OMVs.

There is increasing recognition that the formation of OMVs is a means to transport pathogenic factors from bacteria to host cells, but the mechanism leading to the entry of virulence factors through OMVs is not fully understood yet. The results of our fluorescence-labeled OMV study with confocal microscopy analysis exclude the possibility of direct fusion of OMVs with the plasma membrane of AGS cells, suggesting that OMVs enter gastric epithelial cells by endocytosis. Two major pathways of receptor-mediated endocytosis have been described as (i) lipid rafts/caveolae-mediated and (ii) clathrin-mediated endocytosis. Other endocytic pathways which may be involved are phagocytosis and macropinocytosis which serve to take up either solid particles by formation of large F-actin coated vacuoles, or liquid from the extracellular space, respectively. In this study, we used inhibitors including MDC, a relatively specific blocker of clathrin-mediated endocytosis, Fillipin III, which can block internalization through cholesterol-rich lipid rafts and cytochalasin D, which can obstruct actin polymerization to inhibit macropinocytosis and phagocytosis, to explore the entry of OMVs from *H. pylori* 26,695 strain into gastric epithelial cells. Our results showed that internalization of *H. pylori* OMVs by AGS cells was reduced after the addition of cytochalasin D or a higher dosage of MDC to cells, but it was not affected by Filipin III treatment. Therefore, we suggest that internalization of OMVs from *H. pylori* 26,695 strain depends mainly on macropinocytosis/phagocytosis, and a lesser degree on clathrin-mediated endocytosis. However, it has been suggested that cytochalasin D should be considered a wide-spectrum inhibitor of all internalization pathways because pharmacological inhibition of actin polymerization has been shown to also block endocytosis via clathrin-coated pits and caveolae [46]. Hence, the possibility of involvement of other endocytic pathways in the entry of OMVs from *H. pylori* 26,695 strain cannot be completely excluded. Other studies on the internalization of OMVs isolated from other *H. pylori* strains such as the P12 strain has been reported to involve both clathrin-dependent and clathrin-independent endocytotic pathways [37,38], and the heterogeneous population of OMVs isolated from *H. pylori* 251 strain were found to be internalized via both micropinocytosis and other kinds of endocytosis, mainly dynamin dependent endocytosis [32].

Much research has demonstrated that the virulence factors, such as CagA and VacA, of *H. pylori* are associated with its pathogenesis. Therefore, we originally expected that the contents of virulence factors in bacteria and OMVs may correlate closely with the severity of gastric diseases caused by clinically isolated *H. pylori* strains. However, based on the limited number of clinical strains tested here, a direct relationship between the contents of virulence factors (CagA, VacA and UreA in particular) and the severity of diseases caused by these clinical *H. pylori* strains was not observed, as strains 13,223 and 11,749, which were isolated from patients with much more severe diseases, MALT lymphoma and gastric adenocarcinoma grade III B, respectively, did not contain CagA and VacA (or produced much less) in both bacteria and the corresponding OMVs generated. This result may suggest that some of the phenotypes of these clinical strains could be quite different from those of our laboratory 26,695 strain used; thus, one should be very careful when directly inferring the findings from the laboratory strains on the pathogenicity of *H. pylori* clinical strains. Since the interaction between a host and a pathogen is complicated, the characteristics of *H. pylori* may also be altered during the infection process. The lifestyle and genetic background of the patients with *H. pylori* infection might cause the bacterium to modify itself so that it can adapt to the environment. Therefore, what we observed here might be a result of bacterial adaptation instead of the bacteria’s original characteristics.

In our current report, we found that the OMV production of *H. pylori* 26,695 strain was increased along with the prolongation of bacterial culture up to the early death phase tested. In addition, the amounts of the three major virulence factors CagA, VacA and UreA were continually accumulated during the log phase and reached a plateau after entering the stationary phase. Furthermore, the size of OMVs isolated after 60 h of bacterial growth ranges from 50 to 400 nm in diameter, with an average diameter of 120.20 nm, which is consistent with the size range that has been reported for OMVs from other *H. pylori* strains [30,32]. Previous studies have revealed that a distinct size of OMVs can have different cargo protein compositions and uptake mechanisms [30,31,32], and many factors such as growth conditions, growth phases, environmental stresses and even LPS structure, can also affect the production and size distribution of OMVs and the association of cargo proteins with OMVs [27,33,47,48,49]. OMVs used in this study are collected at a single time point (60 h) of regular bacterial culture and prepared as crude heterogenous populations instead of separating the OMVs according to their sizes, the compositional heterogeneity is inevitable. Therefore, it will be interesting to explore the effect of *H. pylori* OMVs with different sizes and times of collection on the internalization mechanism toward host cells and their pathological outcomes.

OMVs are produced during the growth of bacteria and contain a range of surface antigens and multiple pathogen-associated molecular patterns (PAMPs) that can trigger immune responses [50]. The successful development of *Neisseria meningitidis* vaccine using OMVs has gained increasing attention towards the development of an OMV-based vaccine against other bacterial infections [51]. OMVs from *H. pylori* 7.13 strain were found to induce immune responses in mice without causing mucosal inflammation [52]. Using *H. pylori* 7.13 OMVs as adjuvants with a vaccine derived from outer membrane proteins or whole cell vaccine also significantly improved the protective effect in immunized mice [53]. Notably, LPS or other lipids in OMVs might not have the immunostimulatory role while *H. pylori* 7.13 OMVs were applied as adjuvants [53]. Lipoprotein 20 (Lpp20) located on the surface of *H. pylori* OMVs was previously identified as a vaccine candidate which elicited a strong immune response in mice and the antibody against Lpp20 effectively lowered the colonization of *H. pylori* SS1 strain [54,55]. However, only OMVs, but not Lpp20, were immunogenic in guinea pigs, and immunization with *H. pylori* OMVs did not prevent *H. pylori* G15 strain from colonizing the animals [56], suggesting that the components of OMVs and the differences among species should be taken into consideration during the development of OMV-based vaccines. Based on our unpublished data, we found that the protein profiles of OMVs and outer membrane samples isolated from *H. pylori* 26,695 strain show a certain degree of similarity, indicating that OMVs may be a good candidate for vaccine development. However, our current data showed that these OMVs also carry the major virulence factors CagA, VacA and UreA and were toxic to epithelial cells. Factors that caused the death of epithelial cells treated with *H. pylori* OMVs have still not been clearly identified, and the elimination of key virulence factors alone might not be sufficient to reduce the toxicity caused by OMVs treatment. In addition, these OMVs can also enter gastric epithelial cells within a short period of incubation time, which will reduce the possibility of OMVs to contact with immune cells and elicit immune responses. Therefore, the selection of OMVs as the target for vaccine development is important and the modifications done on OMVs are required to reduce their toxicity and non-immune cell entry.

Studies showed that OMVs from *Pseudomonas aeruginosa* [57,58], *Lysobacter* sp. XL1 [59] and some other Gram-negative bacteria [58] can lyse other bacteria, proposing that OMVs of pathogenic bacteria might be used as biological antibiotics to kill other infectious pathogens. OMVs from *P. aeruginosa* contain peptidoglycan hydrolases (autolysins) and can fuse with the outer membrane of Gram-negative bacteria or adhere to the cell wall of Gram-positive bacteria and lyse the peptidoglycan layer or cell wall [57]. *Lysobacter* sp. XL1 can produce OMVs that have bacteriolytic activity on both Gram-positive and Gram-negative bacteria due to the presence of endopeptidase L5 within the OMVs [59]. These studies suggested that the bacterial killing activity of OMVs requires the fusion of OMVs with other non-self bacteria. In our study, we found that DiI-labeled OMVs isolated from *H. pylori* 26,695 strain did not fuse with the cell membranes of host epithelial cells. Since we did not perform experiments to investigate the interaction of *H. pylori* OMVs with other bacteria, we could not conclude if the OMVs from *H. pylori* have the bacterial killing activity. On the other hand, a recent paper suggested that the lysis and cell death caused by OMVs were mainly targeted to other bacteria rather than the host [60]. However, we discovered that even AGS cells co-incubated with a low dosage of *H. pylori* OMVs (5 μg/mL) could result in 30% cell death. Therefore, the applicability of *H. pylori* OMVs as biological antibiotics against other infectious pathogens requires further investigation.

## 4. Materials and Methods

### 4.1. Bacterial Strains and Growth Conditions

The bacterial strains used in this study are listed in Table 1. *H. pylori* 26,695 wild-type strain (WT, ATCC 700392; CagA^+^ VacA^+^) and clinical strains were first grown on sheep blood (10% *w*/*v*) agar plates containing 10 μg/mL vancomycin (Sigma-Aldrich, St. Louis, MO, USA) and incubated for 48 to 72 h at 37 °C under microaerophilic conditions (5% O_2_, 10% CO_2_ and 85% N_2_). To switch the growth condition from the solid to the liquid media, bacteria were scraped off from the agar plates and resuspended in Brucella Broth media (BD Biosciences, Franklin Lakes, NJ, USA) with 10% fetal bovine serum (FBS, Biological Industries, Kibbutz Beit Haemek, Israel), 1% IsoVitalex (Dr. Plate, Taipei, Taiwan). The flasks were then placed in an anaerobic chamber with shaking at 140 r.p.m and cultivated for up to 72 h under a microaerophilic condition at 37 °C.

### 4.2. Imaging of H. pylori OMV Production with Scanning Electron Microscopy (SEM)

Procedures for obtaining the images of OMV production from *H. pylori* 26,695 strain by SEM were carried out as described previously with some modifications [61]. Briefly, exponentially growing *H. pylori* was subcultured to new blood agar plates and incubated for 1 h at 37 °C under a microaerophilic atmosphere. Then, the silicon slides were placed on the top of *H. pylori* for an additional 30 min of incubation. Subsequently, all sample slides were fixed by placing the slides in 100 mM phosphate buffer (pH 7.2) containing 2.5% glutaraldehyde (Alfa Aesar, Ward Hill, MA, USA) as the primary fixative for overnight at 4 °C, and with 2% osmium tetroxide (OsO_4_) as the secondary fixative for 1 h at 25 °C in the dark. Next, the samples were rinsed twice with 15 min of incubation in 100 mM phosphate buffer (pH 7.2) and then incrementally dehydrated in a series of washes in 50%, 70%, 90%, and 100% ethanol for 10 min each. When the samples were dehydrated to 100% ethanol, a 50%/50% mixture of ethanol/hexamethyldisilazane (HMDS, Alfa Aesar) was placed on the samples, followed by 2 or 3 exchanges of HMDS and immersed in HMDS for 4 h at room temperature. After these exchanges, the samples were kept in a fume hood where HMDS could evaporate off. After that, the samples were coated with gold by an ion-coater device (IB-2, Eiko, Tokyo, Japan) and examined with a SEM (S-4700, Type II, Hitachi, Tokyo, Japan).

### 4.3. Growth Curve Analysis of H. pylori

*H. pylori* 26,695 strain was inoculated with an initial optical density at 600 nm (OD_600_) of 0.1 in Brucella Broth media (BD Biosciences) and incubated with a constant rotation for up to 3 days. The OD_600_ values were measured at various time points.

### 4.4. Isolation of OMVs

*H. pylori* OMVs were isolated from bacterial culture supernatants using a method described by Horstman et al. with some modifications [62]. *H. pylori* 26,695 and clinical strains were inoculated with an OD_600_ of 0.05 and grown in 25 mL Brucella Broth media (BD Biosciences). Various *H. pylori* strains were harvested after 60 h of bacterial growth. After the removal of bacterial cells by centrifugation (4000× *g*, 10 min, 4 °C) in a centrifuge 5810R (Eppendorf, Hamburg, Germany) with an A-4–62 rotor, supernatants were filtered through a 0.45 μm filter and then centrifuged at 200,000× *g* (2 h, 4 °C) in an ultracentrifuge CP80WX with a P70AT rotor (Hitachi) to collect OMVs. Pellets were suspended in 100 μL of 20 mM Tris-HCl buffer (pH 8.1–8.2) and used as the OMV preparation (stored at −20 °C). A bicinchoninic acid (BCA) protein assay kit (Thermo Fisher Scientific, Waltham, MA, USA) with bovine serum albumin (BSA) as a standard was used to measure the protein concentration.

### 4.5. Protein Electrophoresis and Immunoblotting Analysis

The protein concentrations of samples were determined using a BCA protein assay kit. The 4× protein sample dye was added into the samples and the mixtures were boiled for 10 min before gel loading. Equivalent amounts of protein samples (30 μg for OMV samples, 20 μg for dissociation assays and 60 μg for cell lysate samples) were loaded to each lane and separated on 10% sodium dodecyl sulfate–polyacrylamide gel electrophoresis (SDS-PAGE) gels. The resolved gels were stained with 0.25% Coomassie Brilliant Blue R250 (Sigma-Aldrich) reagents or transferred to 0.45 μm nitrocellulose membranes (GE Healthcare, Chicago, IL, USA) with a running current of 350 mA for 2 h. The transferred membranes were blocked with 5% skim milk at room temperature for 1 h, and then incubated at 4 °C overnight with one of the following primary antibodies: rabbit anti-CagA (b-300) polyclonal antibody (sc-25766, Santa Cruz Biotechnology, Dallas, TX, USA) with a 1:3000 dilution, rabbit anti-VacA polyclonal antibody (HPP-5013-9, Austral Biologicals, San Ramon, CA, USA) with a 1:3000 dilution, rabbit anti-UreA (b-234) polyclonal antibody (sc-21016, Santa Cruz Biotechnology) with a 1:3000 dilution, mouse anti-p-Tyr monoclonal antibody (PY99, sc-7020, Santa Cruz Biotechnology) with a 1:500 dilution and mouse anti-GAPDH monoclonal antibody (NB300-211, Novus Biologicals, Centennial, CO, USA) with a 1:10,000 dilution. The membranes were then washed 5 times for 7 min each using PBST (phosphate-buffered saline containing 0.02% Tween-20) or TBST (tris-buffered saline containing 0.02% Tween-20), and incubated with the secondary antibody, IRDye 800CW goat anti-rabbit antibody (LI-COR Biosciences, Lincoln, NE, USA) with a 1:15,000 dilution, IRDye 680LT goat anti-mouse antibody (LI-COR Biosciences) with a 1:15,000 dilution or mouse IgGκ light chain binding protein conjugated to horseradish peroxidase (m-IgGκ-BP-HRP, sc-516102, Santa Cruz Biotechnology), for the detection of p-Tyr with a 1:10,000 dilution at room temperature for 40 min in a light-proof container. The membranes were washed 5 times for 7 min each using PBST or TBST again and scanned using a LI-Cor Odyssey^®^ infrared imaging system (Li-COR Biosciences) or an ImageQuant LAS 4000 mini system (GE Healthcare).

### 4.6. Cell Culture

AGS cells (ATCC 1739, human gastric adenocarcinoma epithelial cell line) were grown in Ham’s F-12 media (Sigma-Aldrich) with 10% heat-inactivated FBS under a humidified atmosphere supplemented with 5% CO_2_ at 37 °C. To cultivate AGS cells for OMV treatment, 100 U/mL penicillin and 100 μg/mL streptomycin (Biological Industries) were also added to the medium. When AGS cells reached approximately 80% confluency, the medium was discarded and AGS cells were washed with PBS once and then subcultured to new 10 cm plates.

### 4.7. Dissociation Assay

Dissociation assay was carried out basically as described previously [63,64,65]. In brief, approximately 20 μg/mL total protein of OMVs isolated from *H. pylori* 26,695 strain were treated with PBS, urea (0.8 M or 8 M), or 1% SDS, respectively (1 h on ice). Samples were then centrifuged at 200,000× *g* (2 h, 4 °C) in an ultracentrifuge CS150NX with a S80AT3 rotor (Hitachi). The resulting supernatants were precipitated with acetone and resolubilized in an equal volume of PBS as the pellets. Both pellets and supernatants were mixed with 4× protein sample dye and subsequently analyzed by 10% SDS-PAGE with immunoblotting detection.

### 4.8. Proteinase Digestion Assays

Proteinase digestion assays were conducted as described previously with some modifications [66,67]. Purified intact OMVs or OMVs that had been first treated with or without sonication (VCX750 Watt ultrasonic processor, Sonics and Materials, Newton, CT, USA) or 1% sodium dodecyl sulfate (SDS) for 30 min on ice were treated with proteinase K (10 μg/mL, Sigma-Aldrich) or trypsin-ethylenediamine tetraacetic acid (EDTA) (10×, Biowest) for 1 h on ice. In the sonication step, OMVs were suspended in 300 μL of PBS and then sonically disrupted for 1 min at setting 1 for 3 s (with an interval of 15 min between each sonication) on ice. All obtained samples were mixed with 4× protein sample dye and subsequently analyzed by 10% SDS-PAGE with Commassie Blue staining and by immunoblotting detection.

### 4.9. Fluorescent Labeling of OMVs

The protein concentration of OMVs was measured using a BCA protein assay kit (Thermo Fisher Scientific). Briefly, OMVs were suspended and incubated in 150 μL of Basic Red 18 (BR-18, Sigma-Aldrich) staining buffer (1 mg/mL BR-18, 200 mM NaCl, 50 mM Na_2_CO_3_, pH 9.2) for 1 h at room temperature [39] or 1% (vol/vol) 1,1′-diocatadecyl-3,3,3′,3′-tetramethylindocar-bocyanine perchlorate (DiI, Invitrogen, Carlsbad, CA, USA) in PBS and incubated at 37 °C for 20 min [68]. Dye-labeled OMVs were collected by ultracentrifugation at 50,000× *g* for 30 min at 4 °C and washed with 0.05 M 4-(2-hydroxyethyl)-1-piperazineethanesulfonic acid (HEPES) (pH 6.8) or PBS until the unbound stain was removed. The pellets containing the fluorescence-labeled OMVs were resuspended in the sample buffer (PBS plus 200 mM NaCl) and stored at -20 °C until later used for up to 4 weeks.

### 4.10. Confocal Microscopy

AGS cells were seeded onto 12-well plates or 3.5-cm glass bottom dishes at 8 × 10^4^ cells/well (dish) under the standard cell culture conditions and allowed to adhere overnight. Cells were stained with fluorescein isothiocyanate (FITC)-wheat germ agglutinin (WGA, 2 mg/mL, Sigma-Aldrich) for 1 h, followed by co-incubation with BR-18-labeled OMVs (100 μg/mL of protein) isolated from *H. pylori* 26,695 strain for 1 h. The supernatants were then discarded and the cells were washed once with PBS. Fixation of AGS cells was carried out by adding acetone (Sigma-Aldrich) and methanol (Sigma-Aldrich) mixture (acetone:methanol = 3:1 in volume proportion) onto the cells for 20 min on ice. After that, the coverslips were washed once with PBS and then mounted with DAPI solution (Invitrogen). Fluorescence was visualized with an LSM510 confocal microscope (Zeiss, Jena, Germany). To investigate the association of OMVs and early endosomes, CellLight^TM^ Early Endosomes-GFP (2 μL/10,000 cells, Invitrogen) was added to the attached AGS cells and incubated for another 16 h. DiI-labeled-OMVs (100 μg/mL of protein) from *H. pylori* 26,695 strain were then added into the cell culture, and placed in a 4 °C refrigerator for 30 min to allow the attachment of OMVs without entering the cells. Live images were taken every 10 min for the first hour and every 30 min onwards using inverted LSM800 (Zeiss) confocal microscopy equipped with an incubation heater.

### 4.11. Flow Cytometry Analysis

The measurement with flow cytometry was adopted from a method described previously with some modifications [69]. Briefly, AGS cells were seeded at 3 × 10^5^ cells/well in 6-well plates under the standard cell culture conditions and allowed to adhere overnight till confluent. The medium was replaced prior to the addition of BR-18-labeled OMVs (100 μg/mL of protein or otherwise stated). After 24 h of co-incubation (or otherwise stated), AGS cells were washed once to remove unbound OMVs and lifted by trypsinization (Trypsin-EDTA 10×, Biowest, Nuaillé, France). Fluorescence measurements were made using a fluorescence-activated cell sorter (FACS) flow cytometer (BD Biosciences) and Accuri C6 software (BD Biosciences). A total of 10,000 events were collected for each sample. Mean fluorescence intensity (MFI) values of cells incubated in the absence of OMVs were subtracted from the values of OMV-treated cells. To determine the proportion of internalized OMVs, extracellular vesicle fluorescence was quenched with trypan blue (0.025% final concentration). Fluorescence was measured before and after the addition of trypan blue to determine the total associated OMVs and intracellular OMVs, respectively. To assess the internalization mediated by lipid raft/caveolae or by clathrin- or actin-mediated endocytosis, cells were pretreated for 1 h with different doses of monodansylcadaverine (MDC, Sigma-Aldrich, 50, 100, 200 or 300 μM), Filipin III (Sigma-Aldrich, 1, 5, 10 or 20 μg/mL) or Cytochalasin D (SI-C2618, Sigma-Aldrich, 1 or 10 μg/mL), and then incubated for 1 h with BR-18-labeled OMVs (100 μg/mL of protein) in the presence of these inhibitors. In all the experimental controls, cells were incubated without any inhibitor for the same period of time. Fluorescence measurements were performed as described above.

### 4.12. Preparation of Total Cell Lysates

AGS cells were seeded at 5 × 10^5^ in 6-cm dishes under the standard cell culture conditions and allowed to adhere overnight. After AGS cells were co-incubated with OMVs (100 μg/mL of protein or other tested concentrations) isolated from *H. pylori* 26,695 strain for 24 h or with *H. pylori* 26,695 bacteria for 6 h, the supernatants were discarded and the cells were washed once with PBS. Cells were collected by trypsinization (Biowest) followed by treating with the lysis buffer (50mM Tris-HCl buffer (pH 7.4), 50 mM dithiothreitol (DTT, Sigma-Aldrich), 1% SDS) to disrupt cell membranes. Additional amounts of 50 mM sodium fluoride (NaF, Sigma-Aldrich) and 0.5 mM sodium orthovanadate (Na_3_VO_4_, Sigma-Aldrich) were added to the lysis buffer to inhibit phophatase for the detection of CagA phosphorylation.

### 4.13. MTT Assay

3-(4,5-cimethylthiazol-2-yl)-2,5-diphenyltetrazolium bromide (MTT, Sigma-Aldrich) assay was used to determine the cell viability. A total of 1 × 10^4^ AGS cells were seeded in each well of 96-well plates and allowed to attach overnight, followed by the co-incubation with bacteria (multiplicity of infection (MOI) = 100) or OMVs isolated from various *H. pylori* strains (100 μg/mL of protein or other tested concentrations). After further incubation with bacteria for 6 h or OMVs for 24 h, AGS cells were cultured in MTT solution (final concentration of 0.5 mg/mL in the cell culture medium) for 2 h. An amount of 100 μL of dimethyl sulfoxide (DMSO, Sigma-Aldrich) was added to each well to dissolve the insoluble MTT product into a purplish solution. The cell viability index was measured at 595 nm using an iMark^TM^ microplate absorbance reader (Bio-Rad, Hercules, CA, USA).

### 4.14. Particle Size Measurement of OMVs

The size distribution of OMVs was analyzed by dynamic light scattering (DLS) using Malvern Zetasizer Nano ZS (Malvern Instruments, Worcestershire, United Kingdom). OMVs isolated from *H. pylori* 26,695 and clinical strains after 60 h of bacterial culture were diluted to a suitable concentration of 4–6 μg/mL with PBS filtered through a 0.22 μm filter before the analysis. The measurements were conducted at 25 °C with 3 runs/sample.

### 4.15. Statistics Analysis

Unless otherwise indicated, each experiment was conducted at least three independent times. Statistical analysis was performed using Excel 2016 (Microsoft, Redmond, WA, USA). Mean ± standard deviation (SD) values were obtained from the representative experiments performed in triplicate. All *p* values were calculated with the Student’s *t*-test using paired, two-tailed distribution. A *p* value lower than 0.05 was considered as significant. * *p* < 0.05, ** *p* < 0.01, *** *p* < 0.001.

## Figures and Tables

**Table 1:** Bacterial strains used in this study and the average diameter of their OMVs. Data are shown as the mean ± SD.

*Helicobacter pylori* Strains	Description	Source	Average Diameter (nm)
26,695	*H. pylori* sequenced strain	ATCC ^1^ 700392	120.20 ± 39.06
13,149	*H. pylori* strain isolated from a patient with gastritis	Division of Gastroenterology, Kaoshiung Medical University Hospital.	89.36 ± 36.75
12,807	*H. pylori* strain isolated from a patient with gastric ulcer		108.10 ± 42.60
13,209	*H. pylori* strain isolated from a patient with duodenal ulcer		92.49 ± 33.48
13,223	*H. pylori* strain isolated from a patient with gastric mucosa-associated lymphoid tissue (MALT) lymphoma		127.40 ± 46.71
10,417	*H. pylori* strain isolated from a patient with gastric adenocarcinoma grade I A		92.64 ± 35.18
11,749	*H. pylori* strain isolated from a patient with gastric adenocarcinoma grade III B		91.75 ± 34.56
11,766	*H. pylori* strain isolated from a patient with gastric adenocarcinoma grade I B		103.20 ± 38.46

^1^ ATCC: American Type Culture Collection.

## Data Availability

Data sharing is not applicable to this article as no new data were created or analyzed in this study.
